# Combining Real and Synthetic Data to Overcome Limited Training Datasets in Multimodal Learning

**DOI:** 10.1101/2025.07.16.25331662

**Published:** 2025-07-17

**Authors:** Niccolo Marini, Zhaohui Liang, Sivaramakrishnan Rajaraman, Zhiyun Xue, Sameer Antani

**Affiliations:** Division of Intramural Research, National Library of Medicine, National Institutes of Health Bethesda, MD, 290894, USA; Division of Intramural Research, National Library of Medicine, National Institutes of Health Bethesda, MD, 290894, USA; Division of Intramural Research, National Library of Medicine, National Institutes of Health Bethesda, MD, 290894, USA; Division of Intramural Research, National Library of Medicine, National Institutes of Health Bethesda, MD, 290894, USA; Division of Intramural Research, National Library of Medicine, National Institutes of Health Bethesda, MD, 290894, USA

## Abstract

Biomedical data are inherently multimodal, capturing complementary aspects of a patient condition. Deep learning (DL) algorithms that integrate multiple biomedical modalities can significantly improve clinical decision-making, especially in domains where collecting data is not simple and data are highly heterogeneous. However, developing effective and reliable multimodal DL methods remains challenging, requiring large training datasets with paired samples from modalities of interest. An increasing number of de-identifed biomedical datasets are publicly accessible, though they still tend to be unimodal. For example, several publicly available skin lesion datasets aid automated dermatology clinical decision-making. Still, they lack annotated reports paired with the images, thereby limiting the advance and use of multimodal DL algorithms. This work presents a strategy exploiting real and synthesized data in a multimodal architecture that encodes fine-grained text representations within image embeddings to create a robust representation of skin lesion data. Large language models (LLMs) are used to synthesize textual descriptions from image metadata that are subsequently paired with the original skin lesion images and used for model development. The architecture is evaluated on the classification of skin lesion images, considering nine internal and external data sources. The proposed multimodal representation outperforms the unimodal one on the classification of skin lesion images, achieving superior performance in every tested dataset.

## 1. Introduction

The increasing availability of multimodal biomedical data [25] is driving the development of novel deep learning (DL) algorithms aimed at analyzing and representing complex medical information, particularly in domains characterized by limited annotated data and high intrinsic heterogeneity in terms of diseases, such as skin lesion classification from dermatology images.

In routine clinical practice, the use of multiple biomedical modalities aids in the capture of complementary aspects of the patient’s health and facilitates the early identification of potentially dangerous diseases [1, 23]. For example, biomedical images provide low-level visual features about disease manifestations, while associated textual reports summarize high-level diagnostic findings identified during the sample examination [14]. Multimodal learning focuses on training DL models integrating samples from various modalities to capture the relationships across different sources of information and to improve the data representations. Therefore, the integration of multiple information sources is particularly advantageous in fields where collecting annotated datasets is time-consuming and data are inherently heterogeneous [24]. Furthermore, the adoption of multimodal learning algorithms is also rapidly increasing [22], helped by the increasing amount of data collected by healthcare systems [1].

However, a significant challenge in the development of multimodal algorithms is that they require sufficiently large datasets with paired samples of the modalities under consideration. Training datasets including paired samples corresponding to the same clinical case are necessary to allow models to associate features across modalities and then to learn cross-modal relationships [2]. An additional complexity is the use of large architectures that are needed to capture relationships among modalities [1, 11]. Large datasets are also necessary to guarantee the robustness and the generalization of multimodal algorithms [16] on unseen data, due to the high dimensionality and complexity of biomedical data, especially considering unstructured samples like images and textual reports. Multimodal learning models, such as foundation models [11], usually include large architectures to capture fine-grained relationships among modalities and therefore require to be trained with large training datasets [16], to avoid overfitting.

Even if the collection of multimodal biomedical data is increasing, some biomedical domains, such as dermatology, still lack large datasets that include samples from multiple modalities. Dermatology focuses on the acquisition and analysis of dermatological skin lesions to identify morphological patterns and features indicative of disease [27], that can be malignant and dangerous (skin cancer is the most common cancer in the United States of America [9, 10]). Despite the large amount of publicly available resources including dermatology images, publicly available datasets lack a large collection of paired samples including images and texts, preventing the development of multimodal architectures. To the best of our knowledge, only a single publication presented a multimodal model that combined dermatology images and reports [29]. However, it includes a private dataset, limiting advances in the field. The metadata linked to images in the publicly available datasets can be exploited to build reports through the use of Large Language Models (LLM) [13]. The large diffusion of LLMs allows both image analysis and automatic generation of reports, but the vast majority of LLMs are not built to analyze medical data and may produce hallucinations, depending on the specific prompt adopted to generate the reports.

The goal of this paper is to exploit image metadata and LLMs to synthesize the corresponding report to train a multimodal architecture, analyzing images and reports. Furthermore, considering the limited amount of training data, annotations are exploited to enrich the data representation. Our proposed architecture generates a robust shared dermatology representation, encoding fine-grained textual concepts within image representations. The transfer of information from reports (highly informative) to images (that are less informative) is known as multimodal co-learning [15], which helps to build more accurate robust data representations [19]. The architecture is tested on the classification of skin lesion images, considering five classes, comparing the performance of the architecture trained only with images and the architecture trained combining information from both modalities.

## 2. Methods

### 2.1. Multimodal architecture

**Figure 1.**
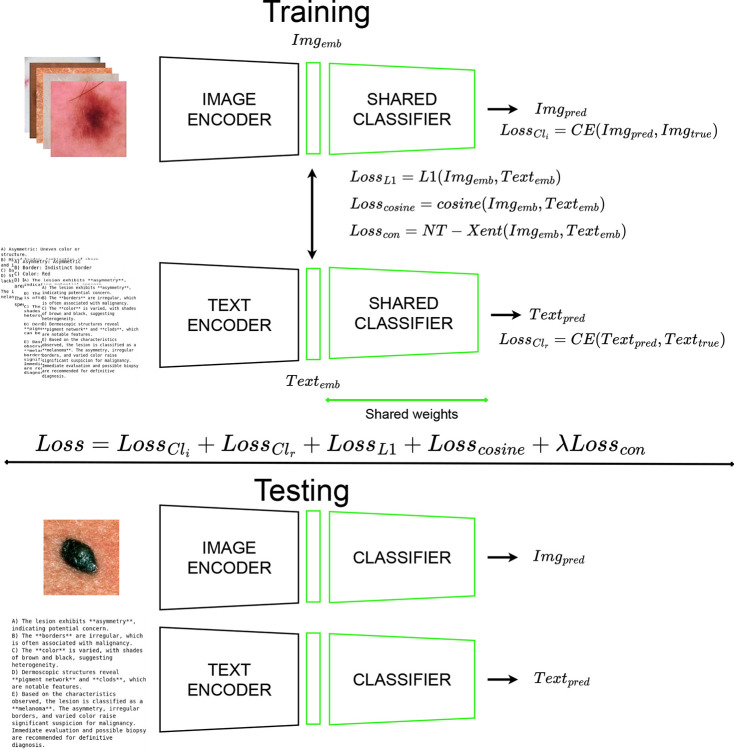
Overview of the multimodal architecture, including with two input branches for skin lesion images and textual reports, followed by a shared projection head and classifier. The training phase requires both modalities with a composite loss combining classification, NT-Xent, L1, and cosine similarity terms to align image and text representations. At inference, each modality can be used independently.

Our proposed multimodal architecture includes two branches to analyze both modalities and a specific training strategy to integrate visual and textual information from dermatology data (paired images and textual reports). It consists of two modality-specific encoders with a shared backbone, including a joint classifier. Here, the encoders produce fixed-size embeddings which feed the shared classifier that processes both types of embeddings. The image encoder is a CNN (i.e. DenseNet121, pre-trained using simCLR algorithm [4]) and the text encoder is a PubMed Bert [8] (pre-trained on PubMed^®^data). The joint classifier is trained to optimize the loss function (i.e. Cross-Entropy) on the class predictions for both images and reports. The choice to share the classifier weights for both modalities is part of the strategy designed to align the modalities. It aids in combining loss functions and the shared classifier weights. Three loss functions are applied to the feature embeddings from both modalities: L1-loss function, Cosine Similarity and a self-supervised (SSL) algorithm (NT-Xent [4] and InfoNCE [17] are tested). Considering the limited amount of training samples, the use of shared weights aims to smoothly align the modalities and to avoid a possible overfitting caused by the three loss functions. The architecture is trained considering two setups, unimodal (i.e. trained with only images), multimodal (i.e. trained with images and reports), while it is evaluated only on the image classification errors (Cross-Entropy loss) during the validation. Figure 1 provides an overview of the multimodal architecture.

### 2.2. Datasets

The data used to develop the architecture include images and textual reports, collected from multiple sources and split into three partitions (i.e. training, validation and testing).

The architecture is trained using both images and reports, while it is tested using only images. The test partition includes only images because the goal of the architecture is to transfer information from reports, that are highly informative, to images that are less informative. Furthermore, only the performance on images is clinically-relevant: a possible performance increase on the assessment of textual reports does not show any advantage in clinical practice, where images are analyzed by experts, that subsequently produce a report.

Data are collected from multiple medical sources to guarantee heterogeneity, in terms of the visual appearance of a disease manifestation, and to test the robustness of the architecture to generalize on data collected from medical sources that are different from the ones used to train the model. Dermatology images include both dermoscopic images [3] (i.e., high-resolution, magnified photographs of skin lesions captured using polarized or non-polarized light) and clinical pictures (i.e., images including skin lesions, usually a non-magnified and non-centered image). Reports are generated from the image metadata, as shown in 2.3.

Data are split in three partitions, as shown in Table 1. The splitting aims to guarantee that training and validation partitions include both dermoscopy and clinical images. Furthermore, the testing partition includes external datasets (i.e. different source than the ones used for training) to assess how the model generalizes on unseen data. The training and validation partitions include data from BCN20000 [5] (dermoscopy, D), Derm12345 [28] (D), Derm7pt [12] (C), DermNet^1^ (clinical, C), DDI [6] (C); the testing partitions include data from BCN20000, Derm7pt, DermNet, derm12345, DDI, HAM10000 [26] (D), SKINL2 [7] (D), Fitzpatrick17k^2^ (C), Hospital Italiano Buenos Aires [20] (D), SD198^3^ (C), PAD UFES 20 [18] (C). The original datasets include many images, but only a subset of the images are selected, corresponding to the following classes: Benign Keratosis (BEK, benign), Benign Nevus (NEV, benign), Actinic Keratosis (ACK, pre-malignant), Basal-Cell Cancer (BCC, malignant), Melanoma (MEL, malignant).

### 2.3. Report generation

The reports are generated by exploiting metadata related to the selected input images. Metadata include information about the class of lesion included in the image, such as melanocytic nevus or melanoma. Some metadata include information that can be linked to one of the five classes selected within the problem (e.g., Melanocytic Nevus is a subclass for Benign nevus, Bowen’s disease is a subclass for Actinic Keratosis). Considering the heterogeneous structure of the metadata, two types of input reports are generated: automatically structured generated and synthesized reports. The automatically structured reports are generated filling a string of text: ”The image includes a *bening* / *malignant* skin lesion, specifically a *class* (specifically a *subclass*), where *bening* / *malignant*, *class*, *subclass* are information collected from the metadata (*subclass* only if available, otherwise it is not added to the report). The synthesized reports are generated using an LLM (i.e. gpt-4o-mini was used). The starting point for the generation is the automatically structured reports, that are filled with additional fields related to the structure of the skin, the color of the image, some dermoscopic structures, and the symmetry/asymmetry of the lesion. Each one of the additional fields include a set of options to be selected, to avoid hallucinations from the LLMs, that are not trained on the specific knowledge requested to analyze dermatology data.

### 2.4. Experimental setup

The experimental setup involves hyperparameter optimization, data encoding, the loss functions adopted to align modalities and data augmentation strategies, and the strategy to handle the class unbalance in the training partition. The hyperparameters have been chosen with a grid search algorithms, focusing on number of epochs (10), learning rate (1e-4), optimizer (Adam), batch size (32), decay weight (1e-5), loss functions weight (1 for all the losses, except the SSL loss functions, which is 0.5 when NT-Xent is used, 0.25 when InfoNCE is used), the temperature of the SSL loss functions (0.5 for NT-Xent, 0.07 for InfoNCE). The data encoding involved the branches to process data modalities: the main experimental choice involves the image encoder, selecting a CNN architecture (i.e. DenseNet121). The choice of a CNN is driven by multiple aspects linked to the need to avoid overfitting: the lack of pre-trained Visual Transformers (ViT) and the limited number of samples cannot guarantee to learn a good image representation on the pre-trained image backbone, in contrast to pre-trained CNN (refined with simCLR pre-training) that can achieve that. The loss functions adopted to align modalities include three elements: L1-loss, Cosine Loss and an SSL (Nt-Xent or InfoNCE) loss function. The first two loss functions are adopted to match the feature embeddings. However, the functions match the representations samplewise within a batch. Therefore, another loss function is adopted (i.e. the SSL loss function). Among the possible choices, NT-Xent and InfoNCE loss were compared. The data augmentation strategy involves both the augmentation of images and reports. The image augmentation strategy includes rotations, horizontal and vertical flipping and a light RGB color augmentation. The report augmentation includes the turnover between the two types of reports, presented in 2.3. Class-wise data augmentation is applied to balance the training dataset: samples from less represented classes are selected more frequently, providing their augmented versions to the model.

## 3. Results

The multimodal architecture outperforms the unimodal baselines in the classification of dermatology images for both internal and external testing partitions and leads to a more robust representation.

Table 2 summarizes the results, reporting the average and standard deviation of Cohen’s *κ*-score across ten independent model repetitions, for every training setup (i.e. unimodal, multimodal, MM). Considering that the data were collected from medical sources different than the ones used to train the model, the results the robustness of the multimodal learning strategy to data distribution shifts.

Both SSL loss functions adopted in the multimodal training strategy achieve higher performance than the unimodal network, although neither emerges as the more robust of the two.

Table 3 summarizes the evaluation of the Silhouette score [21], a metric to assess the quality of a feature representation by measuring how well each sample is clustered, reflecting both intra-cluster cohesion and inter-cluster separation concerning the classes. From the results it is evident that the application of the multimodal training strategy leads to higher scores. Consequently, the multimodal architecture better separates sample classes.

## 4. Discussion & Conclusion

Since biomedical data are inherently multimodal, DL algorithms that integrate multiple modalities can significantly improve clinical decision-making. Even though a growing number of biomedical data sets are becoming available for use with AI algorithms, they tend to be unimodal. We demonstrate the effectiveness of multimodal learning through use of several publicly available skin lesion datasets. However, since they lack annotated reports paired with the images, we present a strategy exploiting real and LLM synthesized data in a multimodal architecture that encodes fine-grained text representations within image embeddings to create a robust representation of skin lesion data.

The results demonstrate that our multimodal training strategy significantly improves classification performance compared to unimodal approaches. This improvement can be attributed to the ability of the model to transfer meaningful information embedded within the textual reports to the image representations. The model benefits from meaningful report information, even though it is synthesized, highlighting the advantages of co-learning strategies in the domain of dermatology, where lesions are highly heterogeneous and annotated data may be scarce. The performance gains were consistent across both internal and external testing partitions, and also considering different imaging types, including dermoscopic and clinical photographs, which are inherently noisier. The findings are also confirmed through the use of the silhouette score, which shows that multimodal representations are more compact and the class dispersion is lower.

Despite the overall performance improvement, the results show considerable variability, as indicated by relatively high standard deviations across experiments, that is not alleviated by the multimodal learning strategy. One possible explanation can be the overfitting on training data, which is only partially addressed by the architectural design.

The proposed multimodal strategy shows great potential in scenarios where large, well-curated datasets with paired modalities are not available. It offers a possibility to bridge the knowledge gap between modalities, allowing models to leverage cross-modal information even under suboptimal data conditions. Future work will focus on further stabilizing the training process and exploring strategies to better align representations across modalities to reduce performance variability and how to exploit the common representation to link knowledge across modalities, without minimal need for strictly annotated data.

## Figures and Tables

**Table 1.** Composition of the dataset collected from multiple sources to guarantee lesion heterogeneity. The dataset includes skin lesions and the synthesized reports and it is split into training, validation and testing partitions. The dataset includes five classes: Benign Keratosis (BEK), Benign Nevus (NEV), Actinic Keratosis (ACK), Basal-Cell Cancer (BCC), Melanoma (MEL).

Dataset	BEK	NEV	ACK	BCC	MEL	Tot
**Training partition**						
BCN20000	796	2944	515	1966	1999	8220
Derm12345	430	1683	16	291	263	2683
DDI	22	22	0	7	5	56
Derm7pt	46	18	0	26	160	250
DermNet	171	39	38	133	62	443
Total	1465	4706	569	2423	2489	11652
**Validation partition**						
BCN20000	170	630	110	421	428	1759
Derm12345	110	455	13	47	55	680
Derm7pt	21	8	0	12	60	110
DermNet	25	7	6	19	10	67
Total	326	1100	129	499	562	2616
**Testing partition**						
BCN20000	172	632	112	422	430	1768
Derm12345	135	534	8	85	82	844
Derm7pt	13	5	0	7	45	70
DermNet	49	11	11	38	18	127
HAM10000	1338	7737	378	622	1305	11380
SKINL2	33	97	0	40	28	198
Fitzpatrick17k	30	39	5	36	115	225
Buenos Aires	88	602	63	340	253	1346
SD198	60	32	62	13	38	205
PAD UFES 20	52	68	13	64	26	223
Total	1970	9757	651	1667	2340	16386

**Table 2.** Performance of the proposed multimodal architecture on skin lesion classification across internal and external test sets. Results include unimodal and multimodal models (with NT-Xent and InfoNCE losses), evaluated via Cohen’s *κ*-score. Statistically significant differences (Wilcoxon test) are marked with (*).

Dataset	Unimodal	MM (NT-Xent)	MM (InfoNCE)
**Internal partition**			
**BCN20000**	0.675±0.015	0.705±0.021*	0.697±0.025*
**Derm12345**	0.731±0.025	0.724±0.013	**0.743±0.013**
**Derm7pt**	0.591±0.052	**0.609±0.041**	0.604±0.066
**DermNet**	0.706±0.049	0.723±0.044	**0.728±0.041**
**External partition**			
**HAM10000**	0.470±0.021	0.483±0.017	0.490±0.012*
**SKINL2**	0.722±0.052	**0.739±0.068**	0.720±0.058
**Fitzpatrick17k**	0.446±0.031	0.463±0.036	**0.464±0.046**
**Buenos Aires**	0.415±0.065	0.422±0.038	**0.442±0.038**
**SD198**	0.492±0.048	**0.550±0.047**	0.499±0.055
**PAD UFES 20**	0.361±0.056	**0.385±0.050**	0.374±0.054

**Table 3.** Overview on the silhouette score evaluated on the test partitions including dermascopies. The evaluation involves the unimodal and the multimodal representation (considering both NT-Xent and InfoNCE as SSL loss function), reporting the average and the standard deviation of the ten models trained.

Dataset	Only Image	MM (NT-Xent)	MM (InfoNCE)
**BCN20000**	0.140±0.014	0.239±0.016	0.262±0.029
**Derm12345**	0.165±0.016	0.255±0.022	0.305±0.027
**HAM10000**	0.126±0.015	0.175±0.022	0.205±0.014
**SKINL2**	0.189±0.022	0.265±0.038	0.280±0.032
**Buenos Aires**	0.045±0.014	0.069±0.009	0.074±0.015

## Footnotes

1 https://www.kaggle.com/datasets/shubhamgoel27/dermnet. Retrieved April 28, 2025

2 https://github.com/mattgroh/fitzpatrick17k, Retrieved April 28, 2025

3 https://huggingface.co/datasets/resyhgerwshshgdfghsdfgh/SD-198, Retrieved April 28, 2025
